# Efficacy and Safety of Tirbanibulin 1% Ointment for Actinic Keratosis at 1-Year Follow-Up: A Real-Life Extension Study

**DOI:** 10.3390/medicina62061012

**Published:** 2026-05-23

**Authors:** Federica Li Pomi, Mario Vaccaro, Michelangelo Rottura, Natasha Irrera, Francesco Borgia

**Affiliations:** 1 Department of Precision Medicine in Medical, Surgical and Critical Care (Me.Pre.C.C.), University of Palermo, 90127 Palermo, Italy; federicalipomi@hotmail.it; 2 Department of Clinical and Experimental Medicine, Section of Dermatology, University of Messina, 98125 Messina, Italy; mario.vaccaro@unime.it; 3 Department of Clinical and Experimental Medicine, Section of Pharmacology, University of Messina, 98125 Messina, Italy; michelangelo.rottura@unime.it (M.R.); natasha.irrera@unime.it (N.I.)

**Keywords:** actinic keratosis, tirbanibulin, tirbanibulin ointment, long-term efficacy, recurrence, real-life, follow-up

## Abstract

*Background*: Tirbanibulin 1% ointment has demonstrated short-term efficacy and excellent tolerability in the treatment of actinic keratosis (AK) on the face and scalp. However, data on long-term efficacy are still lacking. *Materials and Methods*: This prospective, single-center, 12-month extension study included patients with facial and scalp AKs previously treated with tirbanibulin 1% ointment once daily for 5 consecutive days. Long-term analysis was restricted to lesions that had achieved complete clinical and dermoscopic clearance at the 2-month follow-up. At 12 months, the treated areas were reassessed clinically and dermoscopically. High-resolution images obtained at baseline, 2 months, and 12 months were compared lesion by lesion to distinguish sustained clearance, recurrence at the same anatomical site, and the development of new AKs within the treated field. *Results*: Thirty-seven patients were reassessed at 12 months. Of the 228 AKs treated at baseline, 116 lesions had achieved complete clearance at 2 months and were therefore eligible for long-term evaluation. At 1 year, 70/116 lesions (60.3%) remained free of recurrence, whereas 46/116 (39.7%) relapsed. Sustained clearance was observed in 35/51 grade 1 lesions (68.6%), 32/57 grade 2 lesions (56.1%), and 3/8 grade 3 lesions (37.5%). In addition, 35 new AKs developed within the previously treated field. No delayed local or systemic adverse events and no progression to invasive cSCC were observed during follow-up. Patient-reported satisfaction was high, and 94% of patients stated they would be willing to repeat the treatment. *Conclusions*: Tirbanibulin was associated with sustained lesion clearance at one year, particularly in lower-grade AKs. While recurrence remains relatively common—especially in thicker lesions—the treatment was well tolerated and associated with no delayed adverse effects. Its short application regimen and excellent safety profile support tirbanibulin’s role in the long-term management of field cancerization.

## 1. Introduction

Actinic keratosis (AK) is a common cutaneous intraepithelial neoplasm characterized by the proliferation of atypical keratinocytes within the epidermis, primarily induced by chronic ultraviolet (UV) radiation exposure [1]. It predominantly affects older individuals with fair skin phototypes and typically arises on sun-exposed areas such as the face, scalp, and dorsal hands. AK is widely regarded as a precursor to cutaneous squamous cell carcinoma (cSCC), with reported annual per-lesion malignant transformation rates ranging from less than 0.1% to over 16%, depending on histopathological and clinical features [2].

To standardize clinical assessment, Olsen et al. proposed a grading system based on lesion thickness. Grade 1 lesions are slightly palpable and more easily detected by touch than by visual inspection; grade 2 lesions are both visible and palpable, showing moderate thickness, and grade 3 lesions, or hyperkeratotic AKs, present with pronounced keratinization [3].

Although histopathological classification systems have also been proposed, the correlation between clinical thickness and histological severity is not always linear, and Olsen grading remains primarily a practical clinical tool rather than a direct surrogate of biological severity [4,5].

Since AK usually develops within a broader field of actinic damage, therapeutic decisions should address not only visible lesions but also the surrounding field cancerization, to reduce disease burden, recurrence, and the development of new lesions [6,7]. This long-term perspective is clinically relevant, as AK is a chronic and recurrent condition rather than a purely lesion-limited event [6,8].

Current topical therapies include lesion-directed modalities such as cryotherapy and curettage, as well as field-directed treatments including 5-fluorouracil (5-FU) 4% and 5% cream, 5-FU 0.5% plus salicylic acid 10%, imiquimod 3,75% and 5% cream, diclofenac 3% gel in hyaluronic acid, and photodynamic therapy (PDT) [6,9].

While several of these approaches are effective, their use in clinical practice may be limited by treatment duration, local skin reactions, adherence issues, or the need for repeated treatment cycles, making durable clearance and long-term tolerability key therapeutic limitations [6,10].

Among currently available therapies, tirbanibulin 1% ointment (Klisyri^®^, Almirall, S.A., Barcelona, Spain)has emerged as a field-directed option characterized by a short 5-day regimen and a favorable tolerability profile [11,12]. Phase 3 clinical trials demonstrated complete clearance rates of approximately 44% to 54% at day 57 and also included 1-year follow-up data on lesion recurrence after initial complete clearance [11].

Subsequent real-world studies have broadly supported the short-term effectiveness and tolerability of tirbanibulin in routine clinical practice [13,14].

However, long-term real-world data remain limited, particularly concerning the durability of clearance, lesion-level recurrence, and the development of new lesions within the treated cancerization field [15].

The aim of the present study was therefore to assess the 12-month durability of response to tirbanibulin 1% ointment in a real-life cohort previously evaluated at 8 weeks, with specific attention to sustained clearance, lesion recurrence, development of new AKs within the treated field, and long-term safety [16].

## 2. Materials and Methods

### 2.1. Study Design and Setting

This prospective, single-center, 12-month observational extension study was conducted at the Dermatology Unit of the University Hospital of Messina, Italy. The study represents the long-term follow-up of a previously evaluated cohort of patients with AK of the face or scalp treated with tirbanibulin 1% ointment.

All procedures were performed in accordance with the ethical principles of the Declaration of Helsinki and with local institutional requirements. Written informed consent for the use of anonymized clinical data and the publication of clinical images had been obtained from all participants before study inclusion. No directly identifiable patient information was used.

### 2.2. Patient Selection

Patients were eligible for inclusion in the 12-month extension analysis if they met all of the following criteria:

(i) Treatment with tirbanibulin 1% ointment in the parent study;
(ii) Complete clinical clearance of at least one AK at the 2-month post-treatment visit (T1);
(iii) Availability for clinical and dermoscopic reassessment at 12 months (T2).

Patients were excluded if, between T1 and T2, they had received any additional AK-directed treatment within the previously treated field, including either topical or procedure-based therapies. Additional exclusion criteria were the use of systemic immunosuppressive therapy during follow-up, the use of topical corticosteroids at the same site, and the presence of inflammatory, infectious, or neoplastic skin conditions within the treated area that could interfere with lesion identification or outcome assessment.

For the long-term lesion-based analysis, only lesions that had shown complete clearance at T1 were considered eligible for evaluation at T2.

### 2.3. Treatment Protocol

At baseline (T0), all patients had been treated with tirbanibulin 1% ointment applied once daily for 5 consecutive days to the affected area of the face or scalp, according to the approved prescribing instructions. The ointment was applied as a thin layer in an amount sufficient to cover the treatment field, avoiding application to open wounds or damaged skin.

### 2.4. Follow-Up and Outcome Assessment

At the 12-month follow-up visit (T2), patients underwent clinical and dermoscopic examination of the previously treated field. To ensure accurate identification of persistent clearance, lesion recurrence, or new lesions, high-resolution clinical and dermoscopic images obtained at baseline (T0), 2 months (T1), and 12 months (T2) were reviewed systematically and compared side by side.

Lesion outcomes at T2 were classified as follows:

• Sustained clearance: persistent resolution of a lesion that had been documented as completely cleared at T1, as confirmed by clinical examination, dermoscopic evaluation, and image matching across visits.
• Recurrence: reappearance of a clinically and/or dermoscopically evident AK at the same anatomical site of a lesion previously classified as completely cleared at T1, confirmed by direct comparison of serial images and follow-up examination.
• New lesion: development of a new AK within the previously treated field at an anatomical site distinct from those occupied by the baseline lesions.

### 2.5. Safety and Patient Satisfaction

Late-onset local or systemic adverse events were recorded throughout the follow-up period. Patient satisfaction was evaluated at T2 using a 5-point Likert scale (1 = very dissatisfied, 5 = very satisfied) and a binary question (“Yes” or “No”) assessing willingness to repeat the treatment in the future.

### 2.6. Statistical Analysis

Descriptive analyses were performed to summarize baseline demographic and clinical characteristics. Continuous variables were reported as median and interquartile range (IQR), whereas categorical variables were reported as absolute frequencies and percentages.

A multivariable logistic regression model was used to identify predictors of 12-month recurrence based on baseline variables. Since multiple lesions may belong to the same patient, the analysis was conducted at the lesion level, and observations may not be fully independent. Given the sample size and the exploratory nature of the analysis, a standard logistic regression approach was adopted. The prespecified model included the following covariates: age (per 10-year increase), sex, lesion site (scalp vs. face), baseline Olsen grade (grade 2 and grade 3, with grade 1 as the reference category), and a history of cSCC. Odds ratios (ORs) with corresponding 95% confidence intervals (CIs) were calculated for each covariate. All statistical tests were two-sided, and a *p*-value < 0.05 was considered statistically significant.

## 3. Results

### 3.1. Response to Treatment

At baseline (T0), 228 AKs in 38 patients were treated with tirbanibulin 1% ointment once daily for 5 consecutive days. At the 2-month follow-up (T1), 116 lesions (51.0%) had achieved complete clinical clearance and were eligible for long-term lesion-based assessment. At 12 months (T2), 37 patients were reassessed; one patient was excluded because none of the treated lesions had achieved complete clearance at T1 (Table 1).

**Figure 1:**
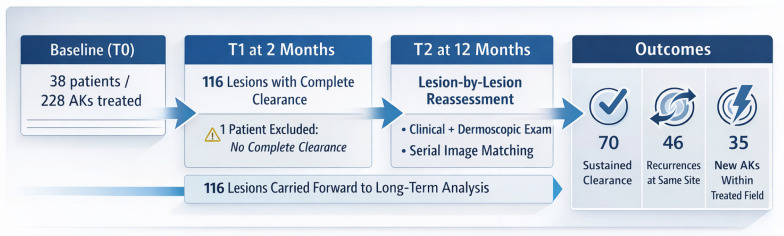
Flow diagram of the study design. At baseline (T0), 38 patients with 228 AKs were treated. At 2 months (T1), 116 AKs achieved complete clearance and were included in the long-term analysis; one patient was excluded because no lesion achieved complete clearance. At 12 months (T2), previously cleared lesions were reassessed by clinical and dermoscopic examination with serial image matching. Final outcomes included 70 lesions with sustained clearance, 46 recurrences at the same site, and 35 new AKs arising within the treated field.

**Figure 2:**
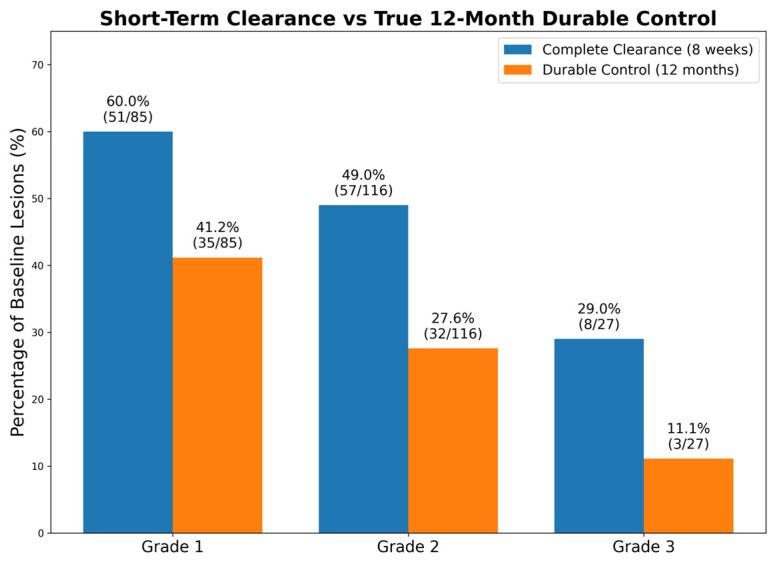
Blue columns indicate the number of lesions with complete clearance 2 months after treatment (T1); orange columns indicate lesions that maintained complete clearance one year after treatment (T2).

Among the 116 initially cleared lesions, 70 (60.3%) maintained sustained clear-ance at T2, whereas 46 (39.7%) showed recurrence at the same anatomical site. Spe-cifically, 68.6% of grade 1 AKs maintained the response, compared to 56.1% of grade 2 AKs and 37.5% of grade 3 AKs (Table 2, Figure 1 and Figure 2).

### 3.2. Regression Model

In the multivariate logistic regression analysis, male sex was associated with a higher likelihood of 12-month lesion recurrence (OR 12.67, 95% CI 2.19–73.25; *p* = 0.005). Conversely, phototype III was significantly associated with a reduced likelihood of recurrence compared with phenotype II (OR 0.20, 95% CI 0.06–0.63; *p* = 0.006).

**Figure 3:**
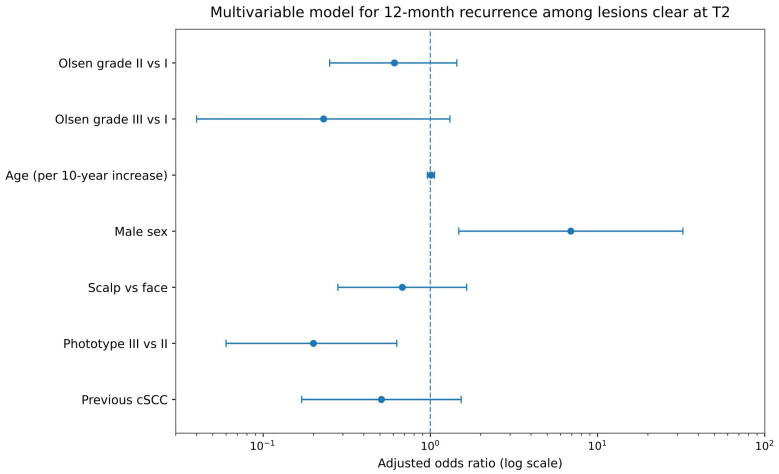
Forest plot of odds ratios from the multivariable lesion-level logistic regression model for 12-month recurrence among lesions with complete clinical clearance at 12 months (T2). Error bars represent 95% confidence intervals based on robust standard errors clustered at the patient level. The dashed vertical line indicates the null value (OR = 1.0).

No statistically significant associations were observed for age (OR 1.01 per 10-year increase, 95% CI 0.96–1.06; *p* = 0.810), scalp location versus face (OR 0.68, 95% CI 0.28–1.65; *p* = 0.390) baseline Olsen grade 2 versus grade 1 (OR 0.61, 95% CI 0.25–1.44; *p* = 0.257), baseline Olsen grade 3 versus grade 1 (OR 0.23, 95% CI 0.04–1.31; *p* = 0.098), or previous history of cSCC (OR 0.51, 95% CI 0.17–1.53; *p* = 0.231) (Figure 3).

### 3.3. New Lesions Within the Treated Field

**Figure 4:**
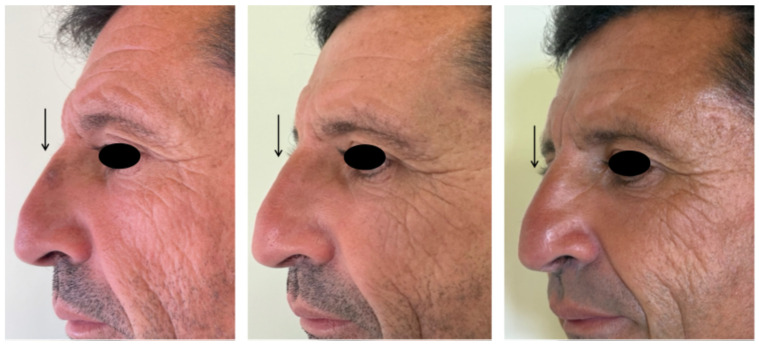
AK on the dorsum of the nose of a male patient before treatment; complete resolution at T1; sustained clearance at T2.

At T2, 35 new AKs were identified within the previously treated field. These lesions were not present at baseline and were classified as: 25 Olsen grade 1, 7 Olsen grade 2, and 3 Olsen grade 3 (Figure 4).

### 3.4. Safety and Tolerability

No delayed local or systemic adverse events were reported during the 12-month follow-up. No cases of progression to invasive cSCC were observed in the treated fields.

### 3.5. Patient Satisfaction

At T2, 37 patients completed the satisfaction assessment using a 5-point Likert scale (1 = very dissatisfied; 5 = very satisfied) and answered a binary question regarding their willingness to repeat the treatment if necessary.

On the Likert scale, 27 patients (73%) rated their satisfaction as 5 (very satisfied), 8 patients (21%) as 4 (satisfied), and 1 patient (3%) as 3 (somewhat satisfied). One patient (3%) assigned a score of 2 (dissatisfied). No patients selected a score of 1 (very dissatisfied).

Regarding the binary question, 35 out of 37 patients (94%) expressed willingness to repeat the treatment, while 2 patients (6%) responded negatively.

## 4. Discussion

AK is a chronic, recurrent keratinocytic dysplasia that develops within field cancerization [9]. Even after complete clearance following treatment, long-term recurrence rates remain substantial. A recent systematic review and pooled analysis of randomized controlled trials reported participant-level recurrence rates of 39–85% and lesion-level recurrence rates of 15–34% at ≥12 months after topical therapies [17].

Accordingly, treatment efficacy in AK should be assessed not only by short-term clearance, but also by durability of response, recurrence patterns, and the development of new lesions within the treated field [1,6].

For tirbanibulin ointment, long-term follow-up data remain limited, as several real-world studies have reported outcomes only up to 2 months [11,18,19,20,21].

To date, the main 1-year follow-up evidence comes from the phase 3 study by Blauvelt et al., in which 124 of 174 patients (71%) who had achieved complete clearance developed at least one AK in the treated area by 1 year; among these patients, 72 of 124 (58%) had recurrence at baseline sites and 52 of 124 (42%) developed new lesions at different sites [11].

In the present real-world extension study, 70 of 116 lesions (60.3%) that had achieved complete clearance at 2 months remained clear at 12 months, whereas 46 lesions (39.7%) recurred, and 35 new AKs developed within the treated field. These findings extend our previous short-term observations and provide clinically relevant information on the durability of response after tirbanibulin treatment.

Our results should be interpreted alongside those of Blauvelt et al., but direct comparisons require caution because the two studies used different analytical frameworks. Specifically, the pivotal trial reported outcomes primarily at the patient level, whereas our study assessed outcomes at the lesion level through side-by-side clinical and dermoscopic matching of the same anatomical sites [11]. This approach allowed a more precise distinction between true recurrence of previously cleared lesions and the emergence of new lesions within the field cancerization.

Notably, sustained clearance in our cohort varied by Olsen grade, occurring in 35 of 51 Olsen grade 1 lesions (68.6%), 32 of 57 Olsen grade 2 lesions (56.1%), and 3 of 8 Olsen grade 3 lesions (37.5%). Although this pattern is clinically consistent with the lower durability generally seen in thicker lesions, baseline Olsen grade was not independently associated with recurrence in the multivariable analysis. This may reflect the limited sample size, particularly for grade 3 lesions.

In broader terms, the recurrence rate observed in our cohort falls within the range reported for other AK therapies. However, these data should not be interpreted as evidence of comparative efficacy. Cross-study comparisons remain inherently limited by the absence of a control group in the present study and by substantial heterogeneity across published reports, including differences in study design, patient populations, treatment protocols, follow-up duration, outcome definitions, and level of analysis. Long-term efficacy data are available for several established AK therapies, including imiquimod 3.75% or 5% cream, 5-fluorouracil 4% or 5% cream, diclofenac-based regimens, and PDT, although study designs and outcome measures vary substantially across reports [22,23,24,25,26,27].

At 1-year, sustained clearance of initially cleared lesions has been reported in 73% of lesions treated with imiquimod 5% cream and 54% of lesions treated with 5-FU 5% cream [22]. In lesion-based follow-up analyses, 85.8% of lesions treated with 0.5% 5-FU plus salicylic acid and 81.0% of those treated with diclofenac 3% gel in hyaluronic acid remained free of recurrence at 12 months [28]. For PDT, the proportion of patients who achieved complete clearance and remained completely clear for at least 12 months was 47% for BF-200 ALA and 36% for methyl aminolevulinate (MAL) [23], while recurrence rates of 19% to 24% have been reported in phase IV follow-up studies of ALA-PDT [24,29].

Within this heterogeneous context, our lesion-level recurrence rate of 39.7% after initial clearance appears clinically meaningful, particularly given the real-world setting and the lesion-by-lesion follow-up design.

The identification of 35 new AKs within the treated field further emphasizes that lesion clearance alone does not fully capture medium-term disease control in patients with field cancerization. In this setting, both recurrence of previously cleared lesions and the emergence of new lesions contribute to the overall disease burden [1,6]. This reinforces the concept that AK management should be viewed as longitudinal field management rather than a single-lesion intervention.

Importantly, no delayed local or systemic adverse events were recorded during the 12-month follow-up, and no features of progression to invasive cSCC were observed in the treated fields. Patient-reported outcomes were also favorable, with 27 of 37 patients (73%) rating satisfaction as “very high” and 35 of 37 patients (94%) reporting willingness to repeat treatment if needed. Taken together, these data support not only the favorable tolerability profile of tirbanibulin, but also its acceptability in routine clinical practice.

Our tolerability data are consistent with the pivotal trials and with subsequent real-world reports, in which tirbanibulin has generally been associated with mild-to-moderate transient local skin reactions, excellent acceptability, and the practical advantage of a 5-day treatment regimen [11,13,18,30,31].

In conclusion, our study provides useful long-term real-world data on tirbanibulin ointment, for which evidence beyond the phase 3 trials remains limited. Its main strengths include the 12-month follow-up of a previously characterized cohort, lesion-by-lesion tracking through combined clinical and dermoscopic image comparison, and the simultaneous assessment of sustained clearance, recurrence, incident lesions, and late safety outcomes. These features add clinically relevant information to the current evidence base on tirbanibulin.

However, the study also has limitations. First, the absence of a control group limits the interpretation of the treatment effect and precludes any direct comparison with alternative field-directed therapies. Therefore, comparisons with other treatments should be considered descriptive and hypothesis-generating rather than comparative. The sample size was limited, particularly for Olsen grade 3 lesions, thereby reducing statistical power. Moreover, because the long-term lesion-based analysis included only lesions that had achieved complete clearance at the 2-month follow-up, the results should be interpreted as reflecting the durability of response among initial responders rather than the overall long-term effectiveness of tirbanibulin. This selection criterion may have introduced selection bias, leading to an overestimation of treatment durability. In addition, although lesion tracking was supported by serial clinical and dermoscopic image comparison, outcome assessment was not blinded. Another limitation of this study is that the analysis was conducted at the lesion level without formal adjustment for intra-patient clustering; therefore, the potential lack of independence among observations should be considered when interpreting the regression results. However, the lesion-level approach enabled precise longitudinal tracking of each lesion via clinical and dermoscopic matching, representing a key strength of the study design.

Further studies with larger cohorts, longer follow-up, and direct comparisons with other field-directed therapies are needed to better define the long-term role of tirbanibulin in the management of AK and field cancerization.

## 5. Conclusions

Tirbanibulin 1% ointment showed sustained lesion clearance at one year, particularly in lower-grade AKs, with recurrence rates falling within the broad range reported for other approved therapies. Its excellent safety profile, minimal local skin reactions, and short treatment duration contribute to high levels of patient satisfaction and adherence. Importantly, most patients reported a willingness to repeat the treatment. These findings may support tirbanibulin as a valuable option for both early and long-term field-directed management of AKs.

## Figures and Tables

**Table 1:** Patients’ and lesions’ characteristics at T2.

Characteristic	Value
Patients included	37
Age, years	71 (66–79)
Male sex	28 (75.7%)
Female sex	9 (24.3%)
Phototype II	10 (27.0%)
Phototype III	27 (73.0%)
Previous cryotherapy	12 (32.4%)
Previous diclofenac 3%	6 (16.2%)
Previous 5-fluorouracil 4%	4 (10.8%)
Previous imiquimod 3.75%	4 (10.8%)
Previous photodynamic therapy	21 (56.8%)
No previous field treatment	12 (32.4%)
History of melanoma	2 (5.4%)
History of cSCC	8 (21.6%)
History of BCC	4 (10.8%)
No previous skin cancer	23 (62.2%)
Eligible lesions	116
Lesions per patient	3 (2–4)
Face lesions	74 (63.8%)
Scalp lesions	42 (36.2%)
Baseline grade 1 lesions	51 (44.0%)
Baseline grade 2 lesions	57 (49.1%)
Baseline grade 3 lesions	8 (6.9%)

**Table 2:** Sustained clearance and recurrence at the 1-year follow-up (T2).

Baseline Olsen Grade	Lesions with Complete Clearance at 8 Weeks	Lesions with Sustained Clearance at 12 Months, (%)	Recurrence at 12 Months, (%)
Grade 1	51	35 (68.6%)	16 (31.4%)
Grade 2	57	32 (56.1%)	25 (43.9%)
Grade 3	8	3 (37.5%)	5 (62.5%)
All grades	116	70 (60.3%)	46 (39.7%)

## Data Availability

All data generated or analyzed during this study are included in this article.
